# Elevated UV photon fluxes minimally affected cannabinoid concentration in a high-CBD cultivar

**DOI:** 10.3389/fpls.2023.1220585

**Published:** 2023-08-11

**Authors:** F. Mitchell Westmoreland, Paul Kusuma, Bruce Bugbee

**Affiliations:** ^1^ Department of Plants, Soils and Climate, Crop Physiology Laboratory, Utah State University, Logan, UT, United States; ^2^ Department of Plant Sciences, Horticulture and Product Physiology, Wageningen University & Research, Wageningen, Netherlands

**Keywords:** cannabis, cannabinoids, UV, ultraviolet photons, photo-protective pigments, specialized metabolism

## Abstract

Ultraviolet photons (UV) can damage critical biochemical processes. Plants synthesize photo-protective pigments that absorb UV to minimize damage. Cannabinoids absorb UV, so increased UV has the potential to increase cannabinoid synthesis. Studies in the 1980’s provided some evidence for this hypothesis in low-cannabinoid cultivars, but recent studies did not find an increase in cannabinoid synthesis with increasing UV in high-cannabinoid cultivars. These studies used low UV photon fluxes, so we examined the effect of higher UV photon fluxes. We used fluorescent UV lights with 55% UV-B (280 to 314 nm) and 45% UV-A (315 to 399 nm). Treatments began three weeks after the start of short days and continued for five weeks until harvest. Established weighting factors were used to calculate the daily biologically effective UV photon flux (UV-PFD_BE_; 280 to 399 nm). Daily UV-PFD_BE_ levels were 0, 0.02, 0.05, and 0.11 mol m^-2^ d^-1^ with a background daily light integral (DLI) of 30 mol m^-2^ d^-1^. This provided a ratio of daily UV-PFD_BE_ to DLI of 41 to 218% of summer sunlight in the field. Cannabinoid concentration was 3 to 13% higher than the control in UV treated plants, but the effect was not statistically significant. Fv/Fm and flower yield were reduced only in the highest UV treatment. These data support recent literature and lead us to conclude that an elevated flux of UV photons is not an effective approach to increase cannabinoid concentration in high-cannabinoid cultivars.

## 1 Introduction

Spectral quality can influence cannabis (*Cannabis sativa* L.) inflorescence yield (Westmoreland et al., 2021) and cannabinoid concentration (Desaulniers Brousseau et al., 2021). Ultraviolet photons (UV; less than 400 nm) interact with multiple photoreceptors to regulate plant development (Wargent et al., 2009a; Wargent et al., 2009b) and promote specialized metabolism (Wade et al., 2001; Schreiner et al., 2012; Coffey et al., 2017).

UV is commonly reported as an energy flux (W m^-2^), but the photon flux (μmol m^-2^ s^-1^) is more useful in photobiology research (Flint and Caldwell, 2003). UV photons are typically categorized into three groups: UV-C (< 280 nm), UV-B (280 to 314 nm), and UV-A (315 to 399 nm) (Coblentz, 1932). Sunlight includes all wavelengths of UV, but UV-C is filtered by earth’s atmosphere. The UV-B photon flux density (PFD) from sunlight is about 0.37% of midday photosynthetic photon flux density (PPFD; 400 to 700 nm), and the UV-A PFD is about 8.5% of peak solar PPFD (Caldwell et al., 1994).

Although solar UV-B PFD is less than UV-A, UV-B photons are higher energy and significantly more biologically effective. Weighting factors to estimate biologically effective UV indicate the relative quantum effect drops by two orders of magnitude between 280 and 315 nm (Flint and Caldwell, 2003).

Many plants produce photo-protective pigments that absorb UV-B photons to protect critical biochemical processes (Schreiner et al., 2012; Coffey et al., 2017). It has been proposed that cannabinoids act as photo-protectants against UV-B (Pate, 1983). Cannabinoids absorb strongly between about 200 and 350 nm (Hazekamp et al., 2005), but this does not necessarily mean UV exposure increases synthesis.

In a highly cited paper, Lydon et al. (1987) reported that THC in flowers of a drug-type variety increased from 2.5 to 3.1% as biologically effective UV-B increased from 0 to 13.4 kJ m^-2^ d^-1^. Notably, there was no effect in a fiber-type variety, which led the authors to conclude that the effect of UV-B on cannabinoid synthesis is “equivocal”, which means uncertain (Lydon et al., 1987). Recent studies have reported that increasing UV-B (Rodriguez-Morrison et al., 2021; Llewellyn et al., 2022) and UV-A (Llewellyn et al., 2022) had no effect on cannabinoid concentration. All studies to date used relatively lower UV photon fluxes, but there is the potential that a higher UV flux would increase cannabinoids.

We sought to determine the effect of increasing UV photon flux up to an exceeding full summer sunlight on yield and cannabinoids of a high-CBD cannabis cultivar.

## 2 Materials and methods

### 2.1 Plant material and environmental conditions

Sixteen rooted cuttings of the chemotype III cultivar ‘Trump’ (T1) were selected for uniformity and transplanted into 6.3 L plastic pots as described by Westmoreland et al. (2021). Plants were pinched to four nodes and grown for ten days in a greenhouse under a vegetative photoperiod (18/6 h light/dark). Plants were irrigated daily with a complete nutrient solution (Westmoreland et al., 2021).

**Figure 1:**
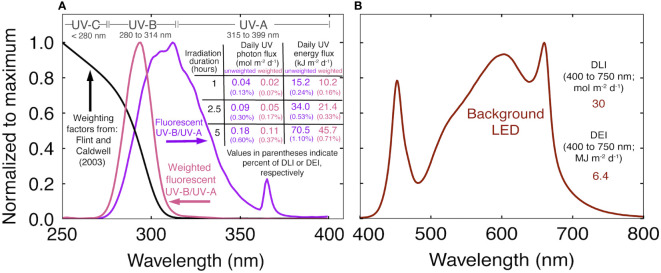
**(A)** Normalized spectral trace of the UV fluorescent light in this study (purple), biological weighting factors from Flint and Caldwell (2003) (black), biologically effective UV spectral trace (pink) and a summary of the UV treatments. The weighted photon and energy flux were calculated by multiplying the unweighted trace by the weighting factors. Values in purple indicate the unweighted daily UV flux. Values in pink indicate the daily biologically effective UV flux. **(B)** Normalized spectral trace of the background white+red LEDs and the daily light integral (DLI) and daily energy integral **(DEI)** during the UV treatments.

After ten days, four plants were randomly assigned to one of four 0.8 m^2^ photon-independent growth chambers with common atmospheric conditions and a reproductive photoperiod (12/12 h light/dark). White+red LEDs (**Figure 1B**) supplied an extended photosynthetic photon flux density (ePPFD; 400 to 750 nm) of 350 ± 30 μmol m^-2^ s^-1^ at canopy height when plants were first moved into chambers. After three weeks, plants grew to a final ePPFD of 700 ± 50 μmol m^-2^ s^-1^ (eDLI: 30 ± 2 mol m^-2^ d^-1^), measured with a full spectrum quantum sensor (Apogee Instruments Inc., model MQ-500). The LEDs contained about 2% far-red photons (700 to 750 nm), so PPFD (400 to 700 nm) was within 2% of ePPFD. White+red LEDs were used because the fixture efficacy is high and, for this reason, are a cost-effective source of photons in commercial cultivation (Westmoreland et al., 2021). Canopy closure occurred in all chambers after three weeks of short days.

Temperature was 26 ± 1 C, measured with six-type E thermocouples per chamber and recorded with a data logger (Campbell Scientific Inc., model CR310). CO_2_ concentration was ambient (~415 ppm) throughout the study. Fans supplied continuous air movement at 1 m per second at canopy height.

### 2.2 UV treatments

UV was supplied with two T5 fluorescent tubes per chamber (**Figure 1A**; AgroMax Pure UV) that supplied an instantaneous UV-B (280 to 314 nm) PFD of 5.5 ± 0.3 μmol m^-2^ s^-1^ (2.2 ± 0.13 W m^-2^) and UV-A (315 to 399 nm) PFD of 4.7 ± 0.1 μmol m^-2^ s^-1^ (1.7 ± 0.04 W m^-2^), for a total UV photon flux (280 to 399 nm) of 10.2 ± 0.6 μmol m^-2^ s^-1^ (4.0 ± 0.2 W m^-2^) at canopy height. Treatments began three weeks after flower induction, once plants had reached a final height, and continued until harvest. Spectral measurements were made before and after each rep with a spectroradiometer (Apogee Instruments Inc., model PS-300). The standard calibration was used for the white+red LEDs. For UV measurements, the spectroradiometer was calibrated with a deuterium arc lamp (StellarNet Inc., model SL3) that provides calibration factors between 200 and 400 nm (Diffey, 2002). UV was applied at the middle of the photoperiod for 0 (control), 1, 2.5, or 5 h. Weighting factors from Flint and Caldwell (2003) normalized at 300 nm were used to calculate daily biologically effect UV photon flux (UV-PFD_BE_; 280 to 399 nm). We report the range of UV-PFD_BE_ from 280 to 399 nm, but the cutoff is arbitrary. After applying weighting factors, UV-A (315 to 399 nm) only accounts for about 2% of UV-PFD_BE_. Biologically effective energy flux, and unweighted photon and energy flux are summarized in **Figure 1A**.

### 2.3 Physiological measurements

Maximum quantum yield of photosystem II (Fv/Fm) was measured every three to four days with a modulated chlorophyll fluorometer (Opti-Sciences Inc., model OS5p+). Measurements were made 30-minutes before lights turned on in the morning, after plants had been dark adapted for 11.5 hours. Five measurements were made per chamber on leaves at the top of the canopy.

Canopy photosynthetic rate was measured on one plant per treatment at harvest in rep two. The system used to make the measurements has been described (Zhen and Bugbee, 2020). Environmental conditions during canopy photosynthesis measurements matched those of the growth compartment at a PPFD of 700 µmol m^-2^ s^-1^, CO_2_ concentration of 400 ppm, and an air temperature of 26 C. Canopy photosynthesis is calculated per m^2^ of ground area. In all cases, the canopy area filled more than 90% of the ground area.

### 2.4 Yield and quality

Plants were harvested 54 days after the start of short days in rep one and 58 days in rep two. Plants were manually separated into leaves, flowers and stems and dried following Westmoreland et al. (2021). Flower yield was calculated as the total flower mass per chamber divided by chamber area. Total yield was the total stem, leaf, and flower mass per chamber divided by chamber area. Roots were not weighed.

A flower sample was harvested from the top of each plant for cannabinoid quantification following Westmoreland et al. (2021). Cannabinoid yield was calculated by multiplying flower yield (g m^-2^) by cannabinoid concentration.

### 2.5 Statistical analysis

The study was a completely randomized block design with two replicates in time as blocks. Data were fit to a linear model and analyzed with regression in R (RStudio, Inc., Boston, MA). Daily UV-PFD_BE_ was treated as a continuous variable. Each chamber consisting of four plants was treated as an experimental unit (n = 2). The average Fv/Fm and cannabinoid concentration of each chamber were used for analysis. Tukey’s Honest Significant Difference test was conducted where results were significant. Canopy photosynthesis measurements were excluded from statistical analysis because only one observation was made in rep two. Effects were considered significant at α = 0.05.

## 3 Results

### 3.1 Yield

**Figure 2:**
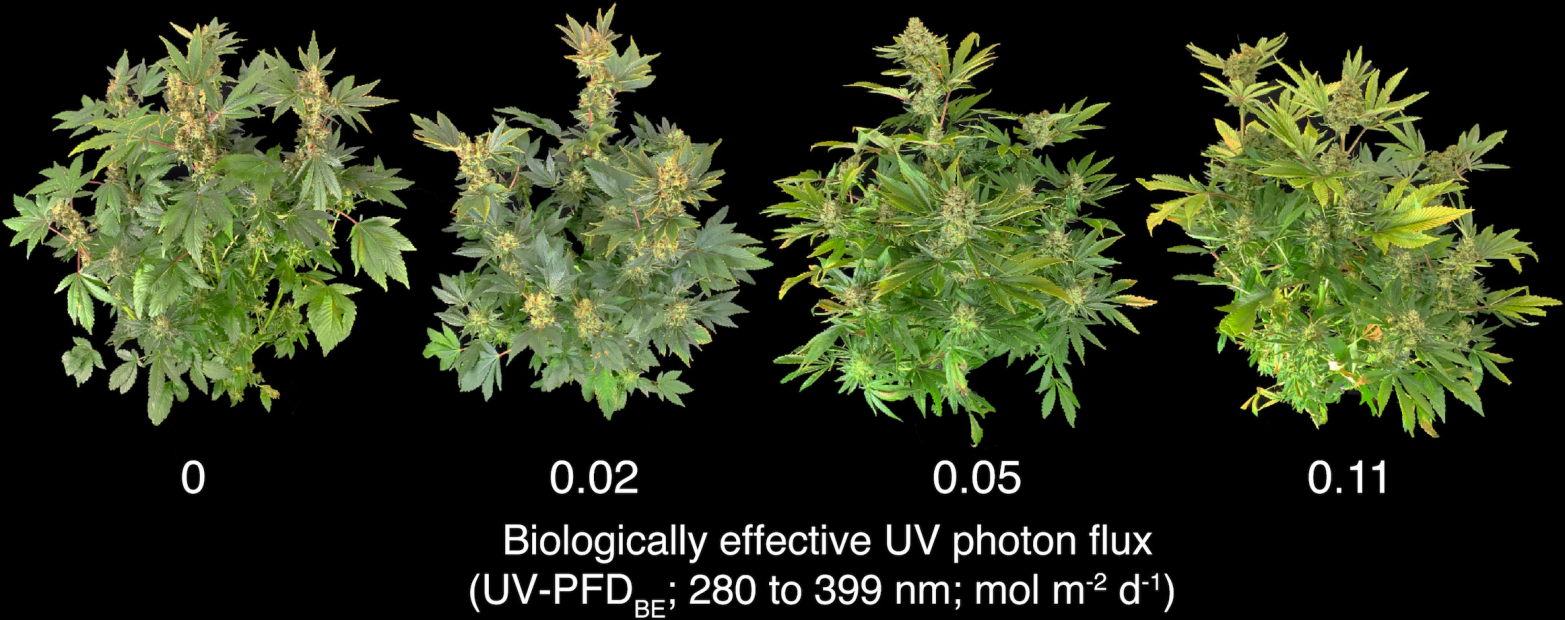
Photo of plants at harvest in rep two. Plants became increasingly chlorotic and damaged with increasing UV-PFD_BE_. UV-PFD_BE_ was calculated using weighting factors from Flint and Caldwell (2003).

The control plants were undamaged until harvest, but treated plants became increasingly chlorotic with increasing daily UV-PFD_BE_ (**Figure 2**). There was no effect of rep on total or flower yield.

**Figure 3:**
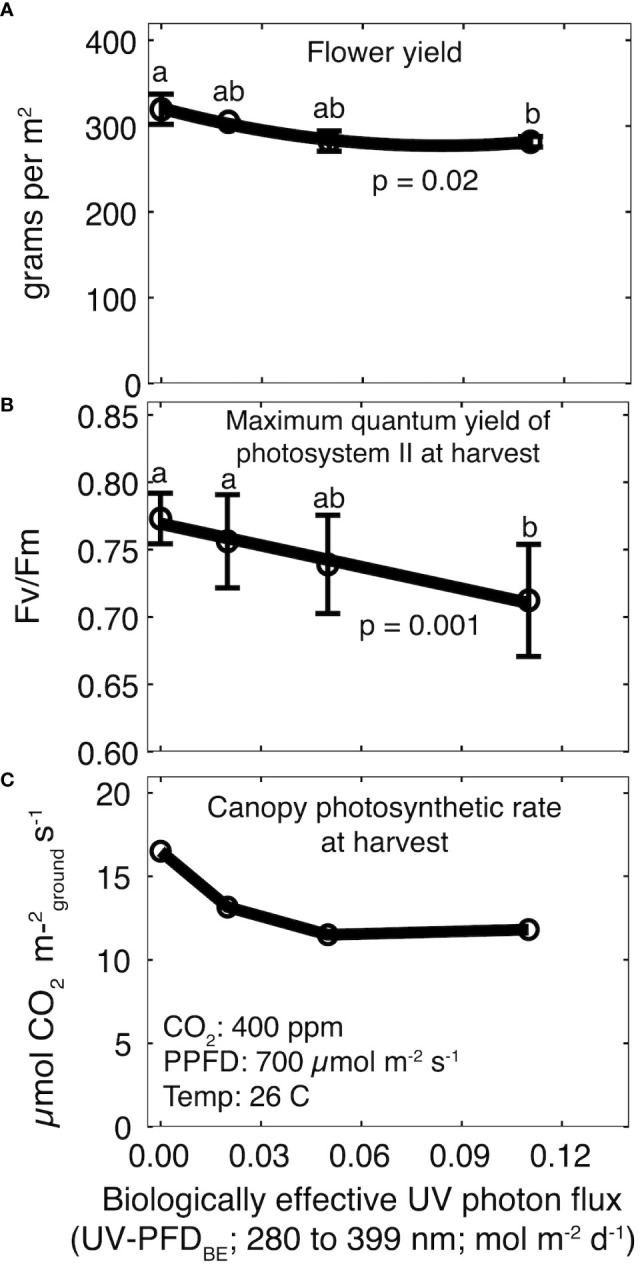
Effect of increasing UV-PFD_BE_ on **(A)** flower yield, **(B)** Fv/Fm at harvest, and **(C)** canopy photosynthesis at harvest. Regression lines are a second-order polynomial model **(A)** or a first-order linear model **(B)** fit to the data. Points with different letters are significantly different according to a Tukey test. Error bars represent the standard deviation between reps (n = 2). No error bars are shown for canopy photosynthesis because measurements were not replicated. UV-PFD_BE_ was calculated using weighting factors from Flint and Caldwell (2003).

Total yield ranged from 586 ± 24 g m^-2^ in the control treatment to 525 ± 23 g m^-2^ at a daily UV-PFD_BE_ of 0.11 mol m^-2^ d^-1^ to (p = 0.05; **Supplementary Figure 1A**), but *post-hoc* analysis did not indicate significant differences among UV treatments. Flower yield was significantly lower than the control at a daily UV-PFD_BE_ of 0.11 mol m^-2^ d^-1^ (p = 0.02), but there were no differences among the control and plants grown at a daily UV-PFD_BE_ of 0.02 or 0.05 mol m^-2^ d^-1^ (**Figure 3A**). Flower yield decreased by 12% from 304 ± 16 g m^-2^ in the control treatment to 268 ± 6 g m^-2^ at 0.11 mol m^-2^ d^-1^. Harvest index (HI; ratio of flower to total yield) was unaffected by UV (p = 0.94). HI was 51 ± 1.2% averaged across all treatments (**Supplementary Figure 1B**).

### 3.2 Cannabinoids

**Figure 4:**
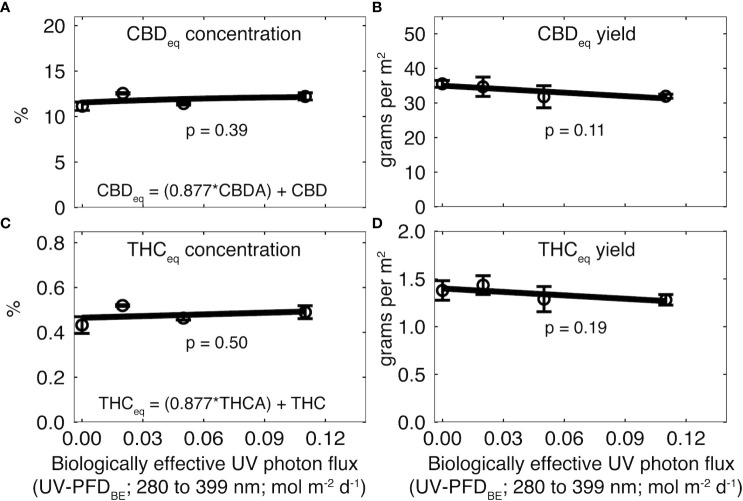
Effect of increasing UV-PFD_BE_ on **(A, C)** cannabinoid concentration and **(B, D)** cannabinoid yield at harvest. Cannabinoid yield was calculated as the product of flower yield and cannabinoid concentration. Cannabinoids were normalized to the mean of rep two. Raw values for each rep are shown in **Supplementary Table 1**. Regression lines indicate a linear model fit to the data. Error bars indicate standard deviation between reps (n = 2). UV-PFD_BE_ was calculated using weighting factors from Flint and Caldwell (2003).

Cannabinoids were higher in rep two than one, so data from rep one were normalized to the mean of rep two. There was no effect of UV on CBD_eq_ (p = 0.39; **Figure 4A**) or THC_eq_ (p = 0.50; **Figure 4C**). CBD_eq_ was 9.0 ± 0.62% in rep one and 11.8 ± 0.58% in rep two. THC_eq_ was 0.32 ± 0.04% in rep one and 0.48 ± 0.03% in rep two (**Supplementary Table 1**).

There was no effect of UV on CBD_eq_ (p = 0.11; **Figure 4B**) or THC_eq_ yield (p = 0.19; **Figure 4B**). CBD_eq_ yield was 25.5 ± 0.9 g m^-2^ in rep one and 33.5 ± 3.5 g m^-2^ in rep two. THC_eq_ yield was 0.91 ± 0.02 g m^-2^ in rep one and 1.35 ± 0.15 g m^-2^ in rep two (**Supplementary Table 1**).

### 3.3 Photosynthesis

There was no effect of rep on Fv/Fm. Fv/Fm was 0.84 ± 0.02 averaged across all treatments before the start of UV treatments and declined in all treatments for the final five weeks (**Supplementary Figure 2**). Fv/Fm at harvest was significantly lower than the control at a daily UV-PFD_BE_ of 0.11 mol m^-2^ d^-1^ (p = 0.001), but there were no differences among the control and plants grown at a daily UV-PFD_BE_ of 0.02 or 0.05 mol m^-2^ d^-1^ (**Figure 3B**). Fv/Fm at harvest ranged from 0.71 ± 0.04 in the highest UV treatment to 0.77 ± 0.01 in the control treatment (**Figure 3B**).

Canopy photosynthesis at harvest was highest in the control treatment at 16.5 μmol m^-2^ s^-1^ and declined to 13.2, 11.5 and 11.8 μmol m^-2^ s^-1^ at a daily UV-PFD_BE_ of 0.02, 0.05 and 0.11 mol m^-2^ d^-1^, respectively (**Figure 3C**).

## 4 Discussion

### 4.1 Cannabinoids were not increased by UV

Lydon et al. (1987) is routinely referenced as evidence that UV radiation increases cannabinoid concentration, but recent studies (Rodriguez-Morrison et al., 2021; Llewellyn et al., 2022), including this one, have shown no beneficial effect of UV on cannabinoid concentration. Cannabinoid concentration in the lowest UV treatment was about 15% higher than the control in rep one and 10% higher in rep two, but the effect was not statistically significant. Based on data from Lydon et al. (1987), it is often proposed that UV only increases cannabinoids in high-THC cultivars. We studied a high-CBD cultivar, but Rodriguez-Morrison et al. (2021) studied two roughly 1:1 CBD : THC cultivars and Llewellyn et al. (2022) studied a high-THC cultivar. Regardless of the chemical profile, UV photons have not been shown to increase cannabinoids in high cannabinoid cultivars. The varieties 40 years ago contained around 3% cannabinoids, less than 20% of modern medical cannabis cultivars. While we cannot dismiss potential interactions among cultivars, the results of Lydon et al. (1987) are not reproducible, likely due to cultivars with relatively low cannabinoid concentrations.

Cannabinoids absorb UV photons, which may lead to degradation. It is possible that UV treated plants synthesized cannabinoids that were degraded by the high-energy UV photons, but it is difficult to draw conclusions from this study. In a separate study in our laboratory, we applied a UV-C (peak ~ 255 nm) dose of 0.01 mol m^-2^ (UV-PFD = 30 μmol m^-2^ s^-1^ for 300 sec) to dry flower that had been ground and spread in a thin layer. Cannabinoids declined by about 15% (unpublished data). UV-C photons are higher energy, and cannabinoids absorb more strongly below 280 nm (Hazekamp et al., 2005), so the effect of UV-B photons on cannabinoid degradation is likely insignificant. Further research is needed to elucidate potential UV-B induced cannabinoid degradation *in vivo.*

There is potential that light sources with different wavelengths or ratios of UV-B and UV-A would lead to an increase in cannabinoids. Llewellyn et al. (2022) used a similar fluorescent UV-B/UV-A light that was used in this study. Rodriguez-Morrison et al. (2021) used narrow-band UV-B LEDs (peak 287 nm). Llewellyn et al. (2022) also demonstrated narrow-band UV-A LEDs (peak 385 nm) did not affect cannabinoid content. Studies from our own laboratory with 405 nm LEDs have confirmed this finding (unpublished data). Based on previous studies, neither UV-B nor UV-A significantly increase cannabinoids in high-cannabinoid cultivars.

### 4.2 Fv/Fm and yield were reduced in the highest UV treatment

Fv/Fm indicates photosynthetic performance, with higher values indicating higher photosynthetic capacity. Fv/Fm of healthy leaves is typically between 0.80 to 0.85 (Sharma et al., 2015). In this study, Fv/Fm at harvest decreased from 0.77 to 0.71 with increasing UV dose. Fv/Fm declined in all treatments after week three, including the control, but the magnitude of decline increased with increase UV. The observed decline in the control treatment is likely related to leaf age, as new vegetative growth stops after three to four weeks of short days. Canopy gas exchange measurements at harvest were not replicated, but they follow the same trend as Fv/Fm and provide a unique insight into the relationship between single-leaf Fv/Fm measurements and whole-plant photosynthesis.

Yield was 12% lower than the control at the highest UV treatment. Fv/Fm and canopy photosynthesis measurements suggest that reduced photosynthesis was the primary cause of the yield reduction at the highest dose, but UV photons can induce upward leaf curling and epinasty which can reduce photon capture and yield. Rodriguez-Morrison et al. (2021) reported upward leaf curling and epinasty at a weighted daily UV photon flux of 0.01 and 0.004 mol m^-2^ d^-1^, respectively. In this study, the two highest UV treatments showed upward leaf curling and epinasty at harvest, thus reduced photon capture may have contributed to the yield reduction in the highest UV treatment. Although the effect on yield was statistically significant, it was a relatively small decrease. This may support the hypothesis that cannabinoids protect from UV-B damage (Pate, 1983).

### 4.3 Relating UV treatments to sunlight in the field

A common criticism of research on UV in controlled environments is that the conditions do not adequately reflect the field (Caldwell et al., 1994). The maximum PPFD from sunlight on a clear day in the middle of summer is about 2000 μmol m^-2^ s^-1^. The UV-B PFD is about 7 μmol m^-2^ s^-1^ and the UV-A PFD is about 160 μmol m^-2^ s^-1^ (Caldwell et al., 1994). In this study, UV-B PFD was about 6 μmol m^-2^ s^-1^, but the UV-A PFD was only about 4 μmol m^-2^ s^-1^. Previous studies used UV-B PFDs ranging from 0.01 to 1.6 μmol m^-2^ s^-1^ and little (Llewellyn et al., 2022) to no UV-A (Rodriguez-Morrison et al., 2021).

Lydon et al. (1987) only reported biologically effective UV-B energy flux (UV-B_BE_ kJ m^-2^ d^-1^) but state the highest flux (13.4 kJ m^-2^ d^-1^) is similar to full sunlight on a weighted energy basis at 0° latitude and 3,000 m elevation. To highlight the magnitude of variability in UV fluxes across the globe, and the complications associated with attempts to replicate field conditions – Caldwell et al. (1994) reported a daily UV-B_BE_ of about 7 kJ m^-2^ d^-1^ from sunlight at 41° latitude and 1450 m elevation.

While the absolute UV PFD is important, the daily UV PFD_BE_ relative to DLI has a larger effect on plant growth (Caldwell et al., 1994). To our knowledge, no values for a daily UV-PFD_BE_ for sunlight have been reported. We developed a model that was confirmed by measurement for daily UV-PFD_BE_ from sunlight. At summer solstice in Logan, UT (41° latitude, 1450 m elevation), the UV-PFD_BE_ is about 0.1 mol m^-2^ d^-1^, which is roughly 0.17% of the DLI. The highest ratio reported in the literature for cannabis is 0.19% (Llewellyn et al., 2022). In this study, the ratio of UV-PFD_BE_ to DLI ranged from 0.07 to 0.37%. This equates to 41 to 218% of full summer sunlight, thus covering a wide range of UV treatments and extending previously reported ranges for cannabis.

## 5 Conclusion

High-cannabinoid cultivars and relaxed legislation have facilitated a reinvestigation into many lingering questions regarding environmental effects on cannabinoid concentration. We found no effect of UV on cannabinoid concentration. Although this study had a small sample size, combined with previous studies, a broad picture is emerging that UV photons do not increase cannabinoid concentration in high-cannabinoid cultivars.

## Data availability statement

The raw data supporting the conclusions of this article will be made available by the authors, without undue reservation.

## Author contributions

Conceptualization, FW, PK, and BB; methodology, BB, PK, and FW; formal analysis, FW; investigation, FW, PK; writing—original draft preparation, FW; writing—review and editing, FW, PK, and BB; supervision, BB; funding acquisition, BB. All authors have read and agreed to the published version of the manuscript.

## Supplementary material

Supplementary Figure 1 Effect increasing UV-PFD_BE_ on **(A)** total mass and **(B)** harvest index. Total mass includes stems, flowers, and leaves. Roots were not weighed. Harvest index is the ratio of flower mass to total mass. Regression lines indicate a linear model fit to the data. Error bars represent the standard deviation between reps (n = 2). UV-PFD_BE_ was calculated using weighting factors from Flint and Caldwell (2003).

Supplementary Figure 2 Time course of Fv/Fm with increasing UV-PFD_BE_. Fv/Fm declined in all treatments after three weeks, but the magnitude of decline increased with increasing UV-PFD_BE_. Error bars represent the standard deviation between reps (n = 2). Statistical analysis was conducted on final Fv/Fm at harvest. UV-PFD_BE_ was calculated using weighting factors from Flint and Caldwell (2003).
