# Supplementary figures and images for: Elevated UV photon fluxes minimally affected cannabinoid concentration in a high-CBD cultivar
Source: Front Plant Sci. 2023 Aug 11;14:1220585. doi: 10.3389/fpls.2023.1220585 (PMC10452874; doi:10.3389/fpls.2023.1220585)

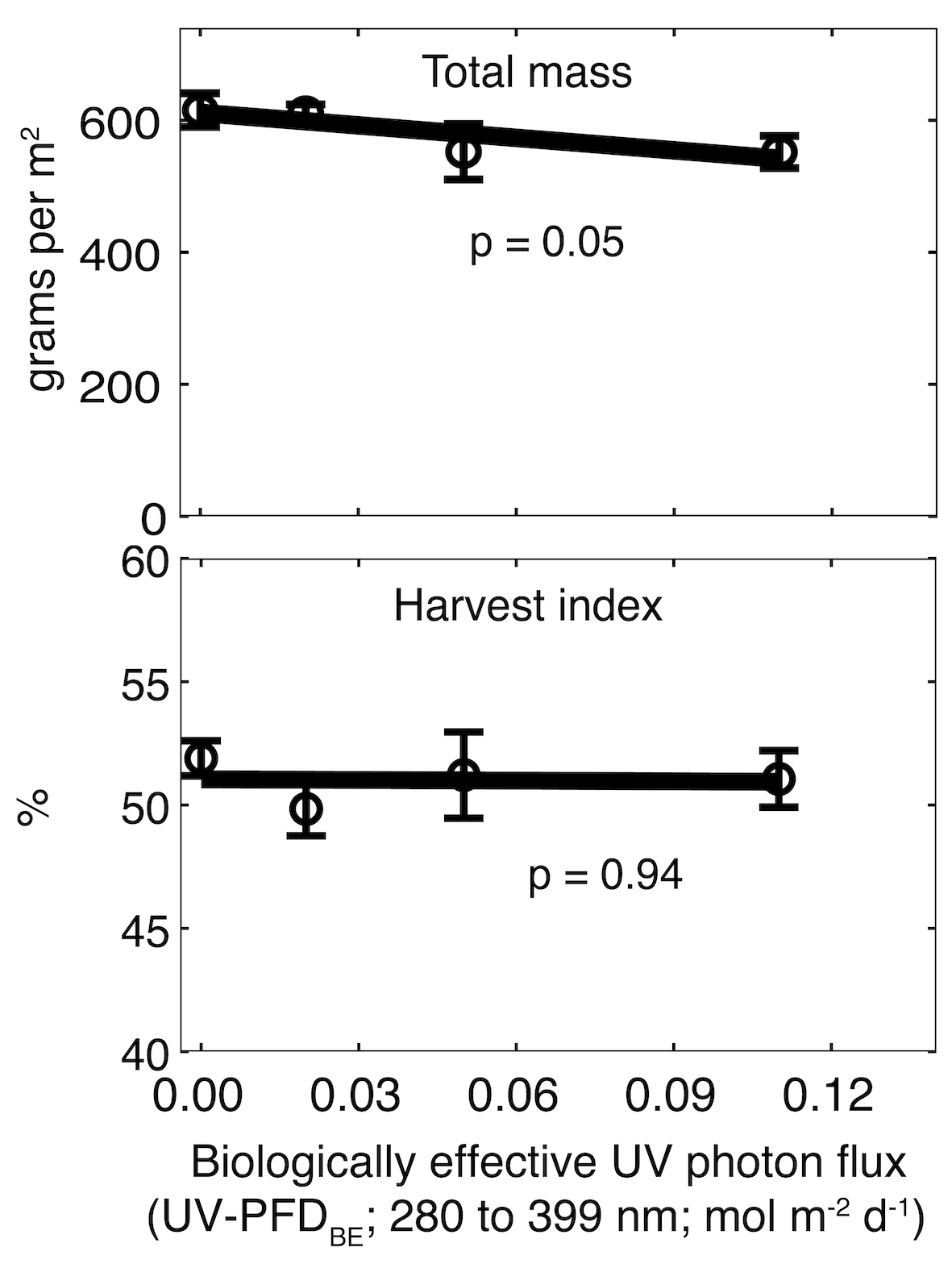

Supplement: Supplementary Figure 1 — Effect increasing UV-PFDBE on (A) total mass and (B) harvest index. Total mass includes stems, flowers, and leaves. Roots were not weighed. Harvest index is the ratio of flower mass to total mass. Regression lines indicate a linear model fit to the data. Error bars represent the standard deviation between reps (n = 2). UV-PFDBE was calculated using weighting factors from Flint and Caldwell (2003). [file Image_1.tif]

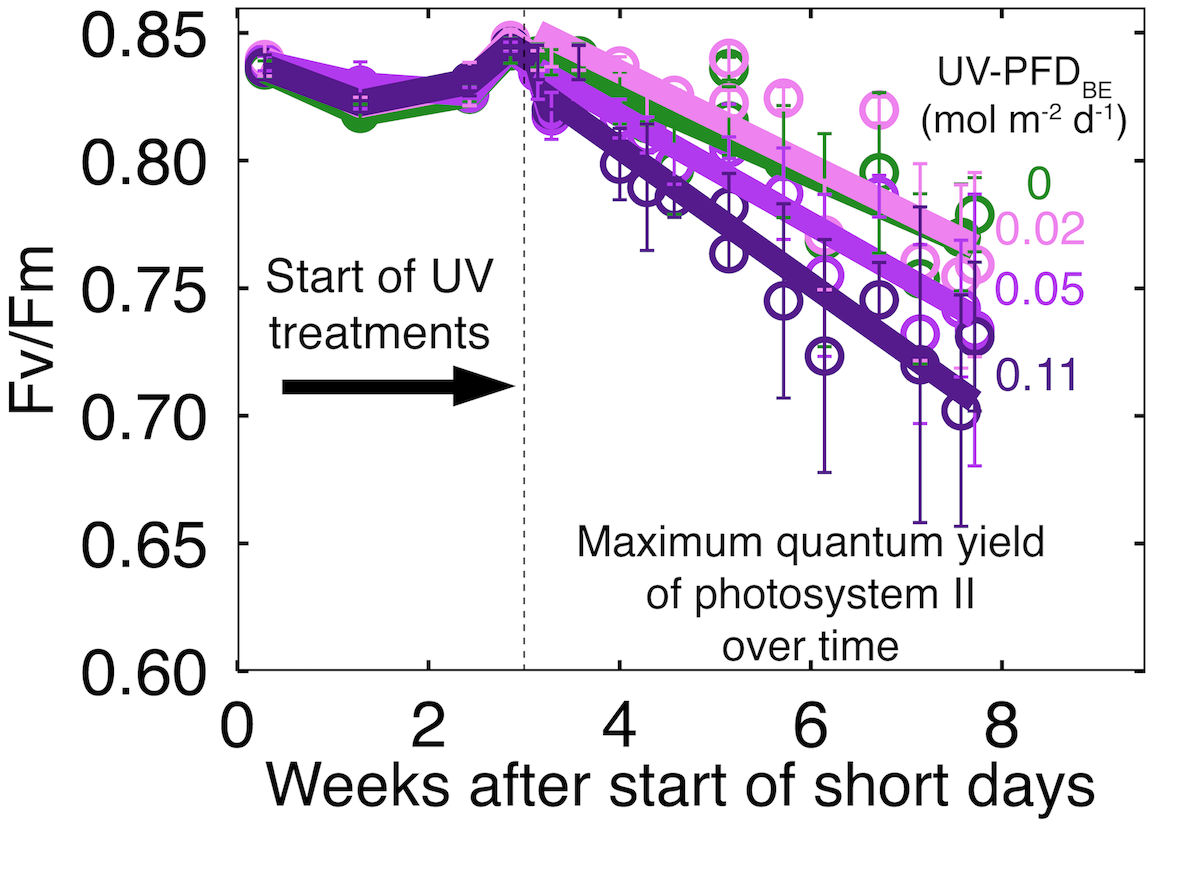

Supplement: Supplementary Figure 2 — Time course of Fv/Fm with increasing UV-PFDBE. Fv/Fm declined in all treatments after three weeks, but the magnitude of decline increased with increasing UV-PFDBE. Error bars represent the standard deviation between reps (n = 2). Statistical analysis was conducted on final Fv/Fm at harvest. UV-PFDBE was calculated using weighting factors from Flint and Caldwell (2003). [file Image_2.tif]
