# Supplementary material for: Elevated UV photon fluxes minimally affected cannabinoid concentration in a high-CBD cultivar
Source: Front Plant Sci. 2023 Aug 11;14:1220585. doi: 10.3389/fpls.2023.1220585 (PMC10452874; doi:10.3389/fpls.2023.1220585)
Supplement: Supplementary file 3 [file Table_1.docx]

**Supplementary Table 1.** Raw CBD_eq_ and THC_eq_ concentration in rep one and two. Values are mean ± standard deviation among plants in each chamber. Rep one was normalized to the mean of rep two for presentation (Figure 4) and statistical analysis. The average concentration of each treatment within a rep was used for statistical analysis (n = 2). CBD_eq_ and THC_eq_ yield were calculated as the product of flower yield and cannabinoid concentration.

| Rep | Daily  UV-PFD_BE_ | ^z^CBD_eq_ | ^y^THC_eq_ | CBD_eq_ yield | THC_eq_ yield |
| --- | --- | --- | --- | --- | --- |
|  | (mol m^-2^ d^-1^) | --------------- % --------------- | | ------------- g m^-2^ ------------- | |
| 1 | 0 | 8.24 ± 0.17 | 0.28 ± 0.002 | 26.5 ± 0.54 | 0.89 ± 0.01 |
|  | 0.02 | 9.52 ± 0.42 | 0.36 ± 0.02 | 24.8 ± 1.10 | 0.93 ± 0.05 |
|  | 0.05 | 8.80 ± 0.75 | 0.32 ± 0.04 | 25.9 ± 2.20 | 0.94 ± 0.11 |
|  | 0.11 | 9.52 ± 0.03 | 0.35 ± 0.004 | 24.6 ± 0.08 | 0.89 ± 0.01 |
|  | All treatments | 9.02 ± 0.62 | 0.32 ± 0.04 | 25.5 ± 0.88 | 0.91 ± 0.02 |
| 2 | 0 | 11.5 ± 0.57 | 0.46 ± 0.03 | 36.2 ± 1.82 | 1.45 ± 0.10 |
|  | 0.02 | 12.6 ± 1.39 | 0.52 ± 0.07 | 36.7 ± 4.05 | 1.50 ± 0.19 |
|  | 0.05 | 11.3 ± 0.71 | 0.46 ± 0.02 | 29.5 ± 1.84 | 1.19 ± 0.06 |
|  | 0.11 | 11.9 ± 0.92 | 0.47 ± 0.04 | 31.5 ± 2.42 | 1.24 ± 0.09 |
|  | All treatments | 11.8 ± 0.58 | 0.48 ± 0.03 | 33.5 ± 3.52 | 1.35 ± 0.15 |
| ^z^ CBD_eq_ = CBD + (0.877*CBDA) | | | |  |  |
| ^y^ THC_eq_ = THC + (0.877*THCA) | | | |  |  |
